# Genomic interrogation of familial short stature contributes to the discovery of the pathophysiological mechanisms and pharmaceutical drug repositioning

**DOI:** 10.1186/s12929-019-0581-2

**Published:** 2019-11-07

**Authors:** Henry Sung-Ching Wong, Ying-Ju Lin, Hsing-Fang Lu, Wen-Ling Liao, Chien-Hsiun Chen, Jer-Yuarn Wu, Wei-Chiao Chang, Fuu-Jen Tsai

**Affiliations:** 1 Department of Clinical Pharmacy, School of Pharmacy, Taipei Medical University, Taipei, Taiwan; 2 Genetic Center, Department of Medical Research, China Medical University Hospital, Taichung, Taiwan; 3 School of Chinese Medicine, China Medical University, Taichung, Taiwan; 4 Laboratory of Bone and Joint Diseases, RIKEN Center for Integrative Medical Sciences, Tokyo, Japan; 5 Graduate Institute of Integrated Medicine, China Medical University, Taichung, Taiwan; 6 Center for Personalized Medicine, China Medical University Hospital, Taichung, Taiwan; 7 Institute of Biomedical Sciences, Academia Sinica, Taipei, Taiwan; 8 Master Program for Clinical Pharmacogenomics and Pharmacoproteomics, School of Pharmacy, Taipei Medical University, Taipei, Taiwan; 9 Department of Medical Research, Shuang Ho Hospital, Taipei Medical University , New Taipei City, Taiwan; 10 Pain Research Center, Wan Fang Hospital, Taipei Medical University, Taipei, Taiwan; 11 Children’s Hospital of China Medical University, Taichung, Taiwan; 12 Department of Biotechnology and Bioinformatics, Asia University, Taichung, Taiwan

**Keywords:** Genome-wide association study, Familial short stature, Single-nucleotide polymorphism, Pharmacogenomics, Drug repositioning/repurposing

## Abstract

**Background:**

Genetic factors, dysregulation in the endocrine system, cytokine and paracrine factors are implicated in the pathogenesis of familial short stature (FSS). Nowadays, the treatment choice for FSS is limited, with only recombinant human growth hormone (rhGH) being available.

**Methods:**

Herein, starting from the identification of 122 genetic loci related to FSS, we adopted a genetic-driven drug discovery bioinformatics pipeline based on functional annotation to prioritize crucial biological FSS-related genes. These genes were suggested to be potential targets for therapeutics.

**Results:**

We discovered five druggable subnetworks, which contained seven FSS-related genes and 17 druggable targerts.

**Conclusions:**

This study provides a valuable drug repositioning accompanied by corresponding targetable gene clusters for FSS therapy.

## Background

Individuals whose body height is in the 3rd percentile or greater below the mean of the population (of the same gender and chronologic age) are defined as short stature (SS). Several mechanisms including endocrine regulation (growth hormone, insulin-like growth factor-1, androgens, and thyroid hormone), proinflammatory cytokines, and paracrine factors have been identified as regulating linear growth [1–3]. Genetic factors account for ~ 80% of variations in human body height [4]. A systematic evaluation of human height genetics through a genome-wide association study (GWAS) uncovered 697 variants, located in 423 loci [5]. Subsequently, those discoveries were extended to rare and very rare variants (with minor allele frequencies [MAFs] of 0.1%~ 4.8%) [6]. In addition, many genetic loci were found to be associated with human height across different populations [7–15], revealing the intricate polygenic architecture that determines human height.

Familial short stature (FSS), also known as “genetic SS”, is found in 23%~ 37% of individuals with SS [16, 17] and is characterized by patients with an SS family history, but normal growth. FSS is one of the most common types of SS and is solely affected by inheritance, thus making it a suitable candidate for identifying genetic loci associated with SS. We can rule out other pathologic causes of growth failure that may potentially confound genetic studies. Based on this idea, an association study of FSS-associated genetic variants in a Taiwanese population was conducted [17]. In that study, six FSS risk genes, including *ZBTB38*, *ZNF638*, *LCORL*, *CABLES1*, *CDK10*, and *TSEN15,* were reported.

Recombinant human growth hormone (rhGH) is currently the only available treatment for SS. However, the efficacy of using rhGH for normal SS remains inconclusive, with some studies showing positive results [18, 19], while others did not [20, 21]. Accordingly, new therapeutics for SS are needed, and new approaches are warranted to expedite treatment. Nowadays, tremendous unveiled genetic loci have been united in tandem with various biological resources and functional annotation methodologies to identify novel drug targets and provide insights for drug repositioning [22, 23]. Hence, genetic loci characterized as being associated with FSS may ultimately be a good starting point for implementation of drug repositioning for SS patients.

In this study, we inquired into the biological and functional links of 122 FSS-associated single-nucleotide polymorphisms (SNPs) in a Taiwanese population and framed an annotation-based analytical pipeline to prioritize FSS-related genes that have the potential to be exploited as drug targets, and appraised the capacity of those drugs to be repurposed.

## Methods

### GWAS analysis of FSS cases and controls

Samples who fulfilled the diagnostic criteria of FSS were recruited from Children Hospital, China Medical University. The FSS was diagnosed by clinicians with the following criteria, including body height less than 3rd percentile to the population with corresponding age, and with a family history of short stature. In addition, only samples with ordinal annual growth rate and coincide bone and chronologic age will be included in this study. The controls in this study were selected from Taiwan Biobank based on their body height, i.e.*,* >75th of all samples. We obtained informed consent from all study participants and guardians. This study was performed in accordance with approved guidelines and regulations.

In sample-level quality control (QC) step, for the 827 FSS patients, we removed 30 duplicated samples, two samples with data quality center (DQC) < 0.82, and 7 samples with call rate < 97%. For the remaining 788 samples, 52 were filtered in kinship QC step and left 736 samples for association analysis. For the controls from Taiwan Biobank, after removing samples with DQC < 0.82, failed plate QC, failed sample QC, missing gender and age information and failed kinship check, resulting in 464 remained for downstream analysis.

In marker-level QC step, for the 628,132 autosomal SNPs, we excluded the SNPs with MAF < 5%, SNP call rate < 98% in either case or control groups, Hardy-Weinberg equilibrium test *p*-value < 0.0001 (based on controls), and with batch effect. The remaining 530,030 (84.38%) SNPs were subjected to association analysis under additive inheritance model.

### Functional annotation of FSS-related SNPs

The region of FSS-associated SNPs (human genome hg19) was annotated using ANNOVAR [24]. The region of variants was categorized as either exonic, intronic, non-coding (nc) RNA intronic, the 5′ untranslated region (UTR), the 3′ UTR, intergenic, upstream, or downstream. For variants located in an exonic region, we further characterized their functional type, i.e.*,* synonymous or non-synonymous.

### Identifying SNPs in linkage disequilibrium (LD) with FSS-related variants

For the 122 FSS-associated variants identified from a GWAS of a Taiwanese population, SNPs that were in high LD to these variants were identified using the 1000 Genome [25] phase 3 database (dbSNP Build 137). SNPs with an *r*^2^ value (a measure of LD) of > 0.8 and within a 100-kilobase (kb) window of FSS-associated variants based on an East Asian (EAS) super-population were selected using the R *proxysnps* package.

### Conspectus of the drug repositioning analysis for FSS

In this study, we proposed a bioinformatics pipeline called SNP-heuristic and expression-based functional unifying network (Shefun) algorithm embodied by two major portions: (1) an SNP-heuristic part and (2) an expression-based functional unifying network part.

The first part is centralized on SNPs. By SNP-based annotations, we could obtain functional states (non-coding/non-synonymous/synonymous), chromatin state, and *cis*-regulation data of each SNP. These data provided two aspects of information for the second part of the Shefun algorithm: resolution of tissue-specificity and determination of “seed” genes. For tissue specificity, based on the enrichment of FSS-associated SNPs with an active chromatin state, we resolved the tissue type(s) for a coexpression analysis. In addition, genes with *cis*-expression quantitative trait locus (eQTL) annotation and/or with non-synonymous variant(s) located in it could be utilized as “seed” genes for network construction.

The second part of Shefun, which mainly focuses on genes, includes several consecutive analytical modus operandi as follows: the construction of tissue-specific expression-based networks; a subnetwork enrichment analysis to establish gene-phenotype relationships; drug repurposing by inferring drug-phenotype relationships; an over-representation analysis; and primary target annotation. All of these functional analyses are unified into a network scene.

### Non-synonymous, chromatin state segmentation and *cis*-eQTL annotations

FSS-associated SNPs (and SNPs in high LD with FSS-related SNPs) were queried in HaploReg (vers. 4.1) [26] using the 1000 Genome Phase 1 database and an Asian (ASN) population. The functional state, chromatin state segmentation (25-state), and *cis*-eQTL information were extracted from the output sheet of HaploReg.

SNPs with a chromatin state of 1~19 were defined as “active”; 20~25 as “inactive”, and the remaining as “not available” (n.a.). For each cell type, we calculated the number of SNPs with an active chromatin state, and calculated one-sided *p* values (*Z* = (*N* – mean(*N*))/SD(*N*), where *N* is the number of SNPs with state 1~19 in the given cell type, and SD is the standard deviation) by comparing to the mean of the number of “active SNPs” across cell types (mean no. = 84.73).

For the *cis*-eQTL part, given the results from chromatin state segmentation, we selected only SNPs with *cis*-eQTL annotation in the following tissue types: whole blood, adipose (subcutaneous) tissues, adipose (visceral omentum) tissues, breast mammary tissue, skin (sun-exposed; lower leg), cells (transformed fibroblasts), muscle (skeletal), skin (not sun-exposed; suprapubic), osteoblasts (prostaglandin E2 (PGE2)), osteoblasts (bone morphogenetic protein 2 (BMP2)), osteoblasts (Dex.) and osteoblasts (untreated). We further merged tissue types into seven categories: adipose, blood, bone, breast, fibroblast, skeletal muscle, and skin.

The SNPs were categorized based on non-coding/non-synonymous/synonymous, the active/inactive chromatin state, and *cis*-eQTL, and visualized them by a radar chart using the R *fmsb* package.

### Genotype-tissue expression (GTEx) transcriptomic dataset pre-processing

GTEx expression data (five tissue types including adipose, breast, fibroblast, skeletal muscle, and skin) were downloaded from *recount2* (https://jhubiostatistics.shinyapps.io/recount/) and processed using the R *recount* package. Samples with an RNA integrity number (RIN) of < 6.0 were filtered. Next, gene expression values were aggregated by the average, and then log_2_-scaled (scaled *E* = log_2_(*E*+ 1), where *E* represents the gene expression value). Then, lowly expressed genes were removed by preserving genes with a scaled expression of > 1 in 80% of the samples in at least one tissue type. Finally, we performed a principal component analysis (PCA) adjustment for latent covariates, also known as surrogate variables, using the R *sva* package.

### Bone tissue dataset pre-processing

As GTEx did not include bone expression data, we thus downloaded a bone biopsy transcriptomic dataset (E-MEXP-1618) of postmenopausal females from ArrayExpress (https://www.ebi.ac.uk/arrayexpress/experiments/E-MEXP-1618/). The raw gene expression values were normalized using the R *gcrma* package.

### Expression-based network construction

The expression-based network (six tissue types, excluding “whole blood”) was consociated with two levels of information: (1) messenger (m) RNA coexpression and (2) protein-protein interactions (PPIs). To do this, for each selected tissue type, FSS-related genes (“seed” genes), constituted by tissue-specific eGenes (from *cis*-eQTL annotation) and genes that contained non-synonymous SNPs, served as input genes for a coexpression network analysis. For each input gene, genes with the top 10/15/20/25/30 highest Pearson’s product-moment correlation coefficient were included to build a subnetwork. Then, the subnetworks were further expanded using PPI information adopted from the Human Protein Reference Database (HPRD, vers. Release9_041310) [27]. Furthermore, self-loops and redundant links were removed from each subnetwork for the sake of conciseness. Different subnetworks were fused into a bigger subnetwork if they contained at least one identical gene.

### Gene set enrichment analysis (GSEA)

The “pathways” for GSEA were the merged expression-based subnetworks, and the gene-level statistics were beta-coefficients (related to “height”) acquired from Taylor et al. (human skeletal muscle biopsies) [28]. The GSEA was conducted using the R *fgsea* package with 99,999 permutations. The significance threshold was set to a false discovery rate (FDR) of < 0.1. The subnetworks that reached a significant threshold were defined as “height-related subnetworks”. For each height-related subnetwork, genes within it were assigned a value of + 1 if the subnetwork was positively enriched (representing a positive “gene-phenotype relationship”) and − 1 if the subnetwork was negatively enriched (representing a negative “gene-phenotype relationship”).

### Ligand/drug repositioning

Ligand-target (gene) interaction data were queried from the Guide to PHARMACOLOGY website (http://www.guidetopharmacology.org/download.jsp, vers. 2019.3). Data were first filtered by the following criteria: (1) human species; (2) non-endogenous agents; (3) a clear type/action of the mechanism for each ligand-target pair; and (4) distinct target (gene symbol) information. We further removed the drug-gene pair of the actions of “binding”, “mixed”, and “neutral”. Next, we assigned a value of + 1 to the ligand-target pair of action of the mechanism of “activation”, “agonist”, “biased agonist”, “full agonist”, “partial agonist”, and “positive”; and also the type of mechanism of “activator” and “agonist”. Similarly, we assigned a value of − 1 to ligand-target pairs with an action mechanism of “antagonist”, “feedback inhibition”, “inhibition”, “inverse agonist”, “irreversible inhibition”, “negative”, “pore blocker”, “slows inactivation”, and “voltage-dependent inhibition”; and mechanism types of “antagonist”, “channel blocker”, “gating inhibitor” and “inhibitor”. Consequently, + 1 or − 1 represents a positive or negative drug-gene relationship, respectively.

For each gene in the height-related subnetworks, the drug-phenotype relationship was inferred by multiplying the assigned values of “drug-gene relationship” and “gene-phenotype relationship”. There were four possibilities to show the logic of how we inferred the drug/ligand effect, i.e., “drug-gene relationship” × “gene-phenotype relationship” = “drug-phenotype relationship”: (1) + 1 × + 1 = + 1; (2) + 1 × − 1 = − 1; (3) -1 × + 1 = − 1; and (4) -1 × − 1 = + 1. A final value of + 1 suggests that the drug may enhance or exacerbate the phenotype of interest, and a final value of − 1 suggests that the drug may alleviate, diminish, or inhibit the phenotype of interest. The repositioning analysis revolved around genes in height-related subnetworks, and drugs/ligands were selected which possibly targeted those genes with a calculated value (drug-phenotype relationship) of + 1 only, as this meant that the selected drugs/ligands possibly enhanced the phenotype of interest (i.e., height) and therefore was a potential candidate for repurposing to FSS.

### Gene ontology (GO) biological process (BP) terms and Kyoto encyclopedia of genes and genomes (KEGG) pathway over-representation analysis (ORA)

Height-related subnetwork genes were subjected to a GO analysis [29] to assess their enrichment in BP terms. The enrichment test was performed using “weight01” implemented in the R *topGO* package. Moreover, the KEGG ORA test was performed using the R *clusterProfiler* package. The Benjamini-Hochberg (BH) method was applied for multiple test corrections.

### Statistical and bioinformatics analysis

All in-house statistical and bioinformatics scripts for drug repositioning analysis were written in R language (https://www.r-project.org/). Gene symbols from different sources were unified using the R *HGNChelper* package. The conversion between gene symbols, Entrez Gene ID, and Ensembl Stable ID was performed using the R *clusterProfiler* package. The networks were illustrated using the R *igraph* package utilizing the Fruchterman-Reingold (FR) algorithm.

## Results

### Genome-wide association and genotyping approaches reveal a total of 122 FSS-associated SNPs

To determine novel susceptible genetic loci of FSS, FSS patients (*n* = 788, male = 51.91%) from Children’s Hospital, China Medical University were enrolled. The diagnosis of these patients (cases) was made by clinicians according to the diagnostic criteria of FSS **(**Additional file 1**: Fig. S1)**. The patients with growth hormone deficiency were excluded from this study. The controls (*n* = 435, male = 42.67%) were from Taiwan Biobank that whose height was above the 75th (Q3) of the total population. Both cases and controls were Han Chinese population residing in Taiwan. After sample–level and marker-level quality control, 530,030 SNPs were subjected to initial genome-wide association screening under the additive inheritance model. Multidimensional scaling (MDS) was performed and no significant population stratification was found (Additional file 2**: Fig. S2**). As shown in Additional file 3**: Fig. S3**, significant associations between genetic loci and FSS were observed. In total, we identified 14 genome-wide significant (*p* < 5 × 10^− 8^) SNPs in the genome-wide screening of FSS cases and controls (Additional file 6**: Table S1**), including rs822611 (Chr 1), rs6731651 (Chr 2), rs16828530 (Chr 3), rs9290657 (Chr 3), rs10028040 (Chr 3), rs1863593 (Chr 8), rs16900402 (Chr 8), rs28786672 (Chr 9), rs7852806 (Chr 9), rs2172912 (Chr 12), rs12826453 (Chr 12), rs9520911 (Chr 13), rs17732181 (Chr 17), and rs4815179 (Chr 20). In present study, we also identified the top 88 genetic loci (Additional file 6**: Table S1** with *p <* 10^− 4^). These 88 novel genetic loci were located in the 44 closest genes. Among these 44 closest genes, eight genes have at least two SNPs within the same gene. These eight closest genes included *AGO4*, *SESTD1*, *PARD3B/ICOS*, *RFC1*, *UNC5C*, *IL7*, *BCL11B*, and *MIAT/MN1*. Among them, *BCL11B*, *IL-7*, *MN1*, and *UNC5C* are involved in embryonic, connective tissue, organ development, and developmental disorders.

Moreover, our previous study suggested 34 SNPs that were also associated with an FSS risk [17]. These 34 human height-related SNPs were located in the 13 closest genes. These 13 closest genes included *TSEN15*, *EFEMP1*, *ZNF638*, *CEP63*, *ZBTB38*, *LCORL*, *HHIP*, *ANAPC10*, *GSDMC*, *QSOX2*, *ADAMTSL3*, *CDK10*, and *CABLES1* that also involved in embryonic, organismal, and tissue development.

### Functional annotations of 122 FSS-associated SNPs

**Fig. 1:**
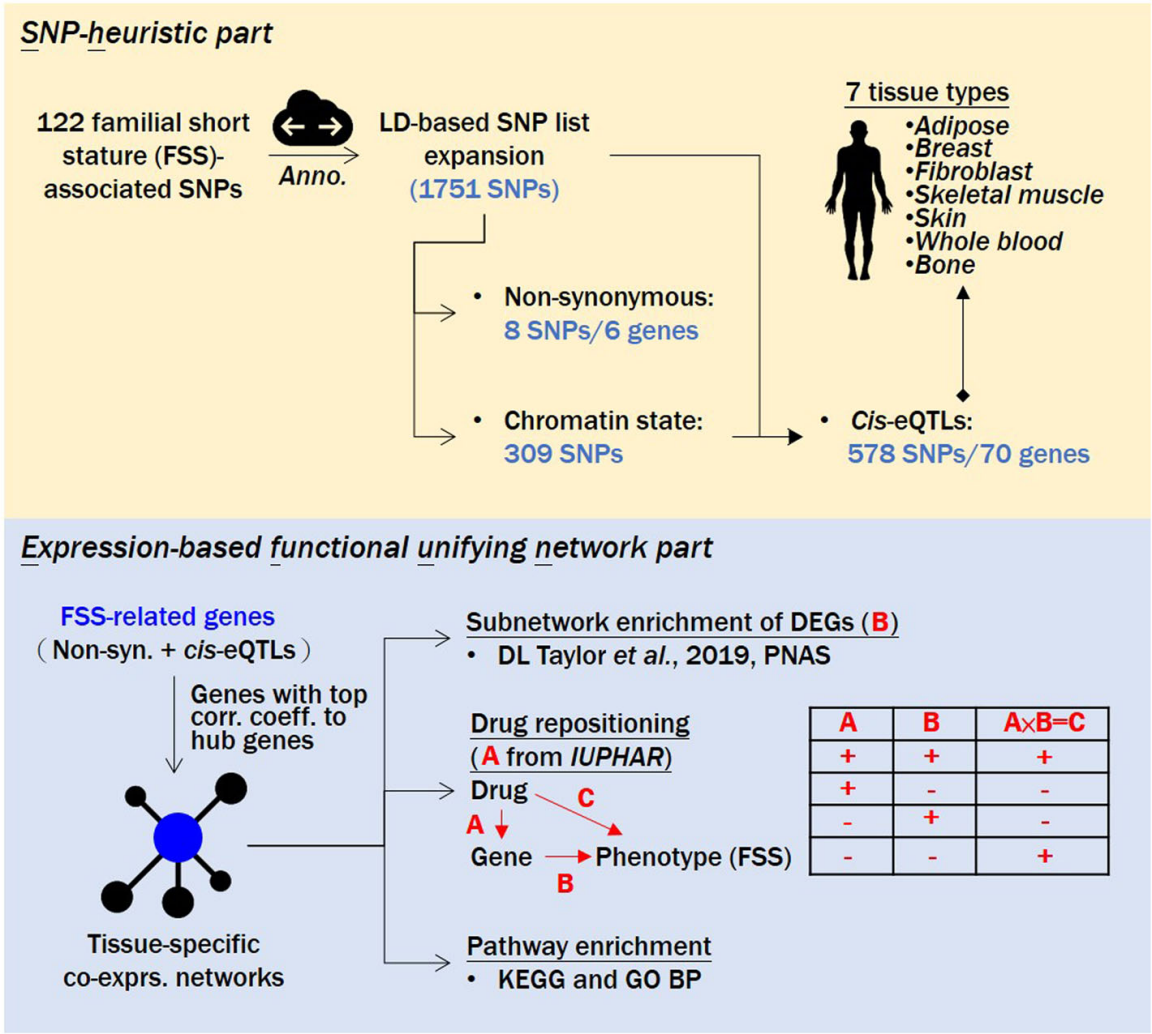
Schematic showing an overview of the drug repositioning pipeline in this study

To identify input genes for the downstream analyses, we consolidated several SNP annotation criteria to map the SNPs to genes (Fig. 1 **[top]**). In the 122 FSS-associated SNPs, most were located in intronic (*n* = 53, 43.44%) and intergenic (*n* = 58, 47.54%) regions (Additional file 7**: Table S2**). Among 122 SNPs, four SNPs were located in an exonic region (Additional file 8**: Table S3**).

**Fig. 2:**
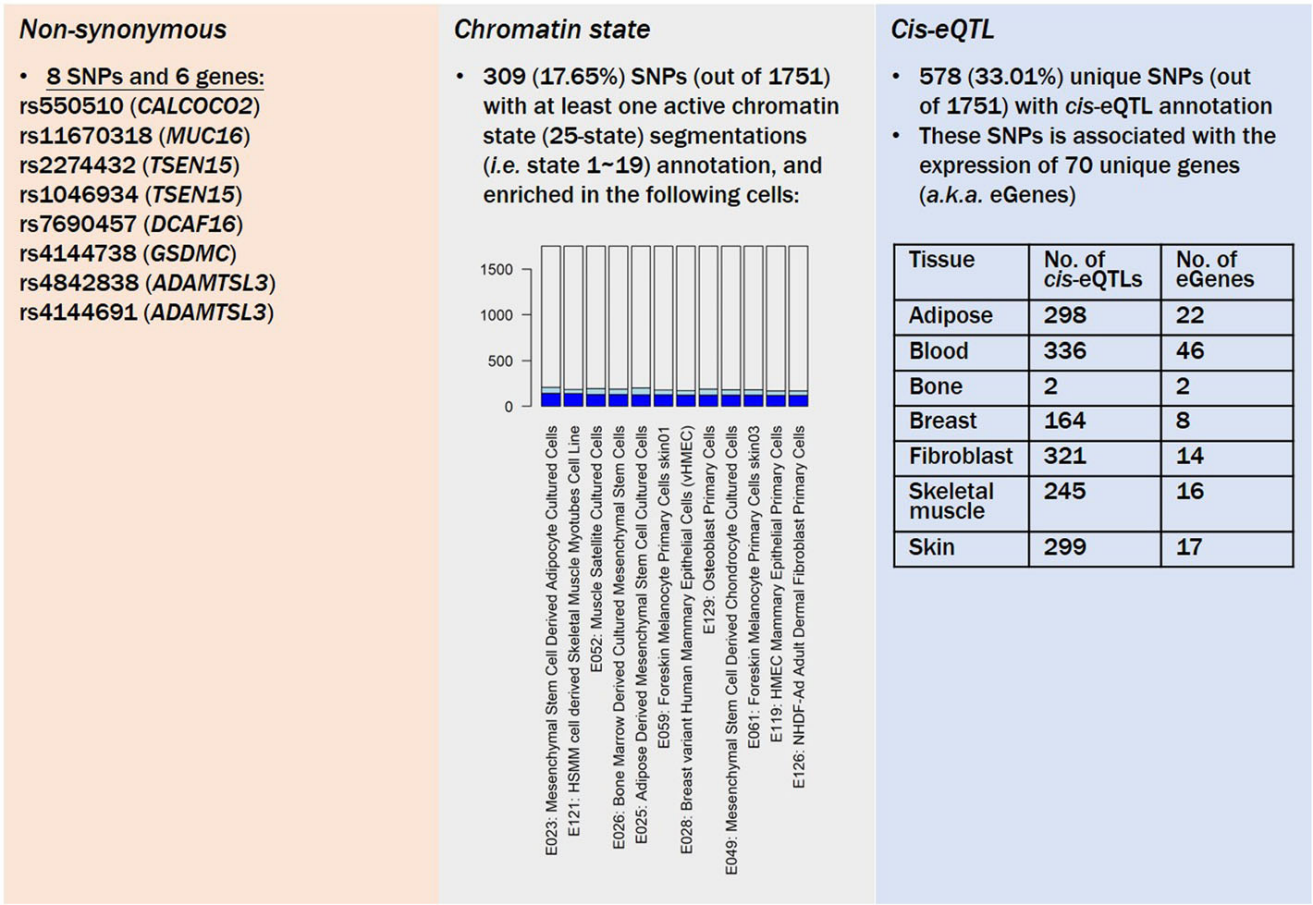
Schematic showing results of the single-nucleotide polymorphism (SNP)-heuristic part analysis. The barplot in the middle panel shows cell types that were significantly enriched in SNPs with an active chromatin state. Blue color indicates SNPs with chromatin state segmentation of 1~19; the light-blue color indicates SNPs with chromatin state segmentation of 20~25; while the remaining have no available annotations

As GWAS and genotyping approaches selected the genotyped SNPs using an LD-tagging method, it could potentially miss causal SNPs that are linked to FSS. Therefore, we expanded the SNP list by querying SNPs in high LD (*r*^*2*^ > 0.8 within a 100-kb window) with our SNP list using the 1000 Genome (phase 3, vers. 5a) EAS database, resulting in 1751 SNPs (121 FSS-associated SNPs and 1630 SNPs in LD with FSS-associated SNPs, where rs10086016 was excluded due to a lack of gene annotation). With the expanded SNP list, we next queried their (1) exonic function, (2) chromatin state segmentation (25-state), and (3) *cis*-eQTL information using HaploReg (vers. 4.1) (Fig. 2).

As a result, we identified six genes (*CALCOCO2*, *MUC16*, *TSEN15*, *DCAF16*, *GSDMC*, and *ADAMTSL3*) in which eight non-synonymous SNPs were located (Fig. 2 **[left] and** Additional file 9**: Table S4**). In addition, among 1751 SNPs, we found 309 (17.65%) SNPs with at least one active chromatin state segmentation (states 1~19) annotation. These SNPs were enriched (*p* < 0.1) in different cell types including adipocytes, skeletal muscle cells, bone marrow-derived cells, skin melanocytes, mammary epithelial cells, and bone-related cells such as osteoblasts and chondrocytes (in total 16 cell types, with brain-related cell types excluded; Fig. 2 **[middle],** Additional file 4**: Fig. S4, and** Additional file 10**: Table S5**).

**Fig. 3:**
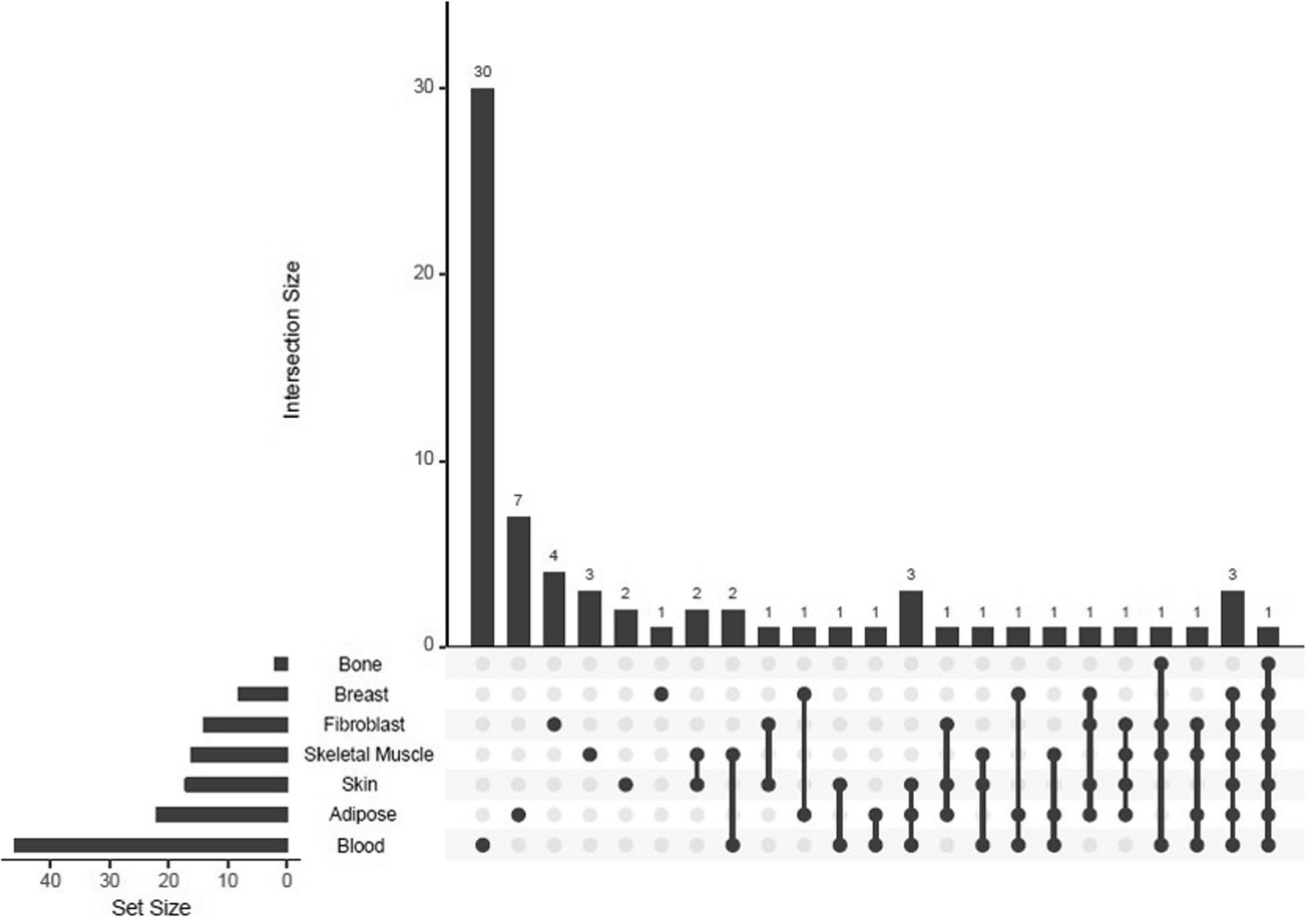
Schematic showing the number of intersections of genes in seven tissue types

Based on these findings, we focus on seven tissues including adipose, blood, bone, breast, fibroblast, skeletal muscle, and skin to seek SNPs with *cis*-eQTL annotation, and identified 298 (17.08%), 336 (19.19%), 2 (0.11%), 164 (9.37%), 321 (18.33%), 245 (13.99%), and 299 (17.08%) *cis*-eQTLs, respectively. In total, these 578 (33.01% of 1751) *cis*-eQTLs were correlated to 70 unique eGenes. In greater detail, the numbers of eGenes in each tissue type were 22, 46, 2, 8, 14, 16, and 17, respectively (Fig. 2 **[right] and** Additional file 5**: Fig. S5**). However, the number of eGenes shared among different tissues was relatively low (Fig. 3), suggesting the uniqueness of the SNP-gene regulation machinery.

**Fig. 4:**
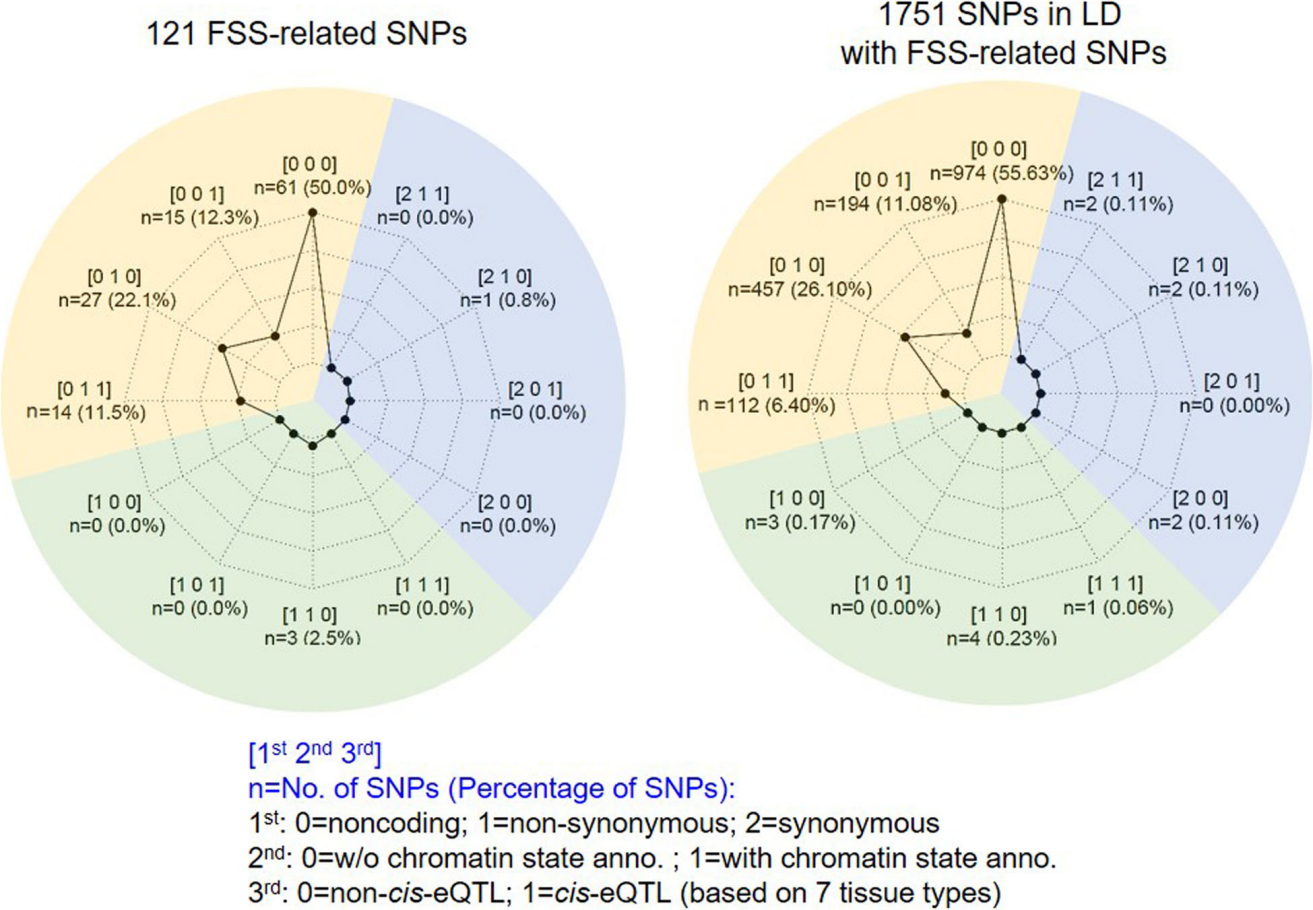
Radar charts showing the number and percentage of the annotation status from 121 familial short stature (FSS)-associated single-nucleotide polymorphisms (SNPs; left panel) and 1751 SNPs (right panel)

Overall, we categorized the SNPs based on annotations, including the functional state (non-coding/non-synonymous/synonymous), chromatin state segmentation (25 states), and *cis*-regulation (Fig. 4).

### Construction of expression (mRNA-coexpression and PPI)-based networks

Given the hypothesis that genes collaborate together to form functional units and to regulate a specific phenotype/pathology (in this case, FSS), we next utilized two published transcriptomic datasets (GTEx [vers. 7] for adipose, breast, fibroblast, skeletal muscle, and skin tissues; and E-MEXP-1618 for bone tissue) to capture the cooperating unit by constructing a so-called “expression-based network”.

To do this, FSS-related genes (composed of tissue-specific eGenes and genes with a non-synonymous annotation) served as “seed” genes for network construction. For each tissue type, we created a network by calculating Pearson’s product-moment correlation coefficients between each of the “seed” genes and the other genes. To focus on the most relevant coexpression links and also to take network robustness into consideration, we identified the top 10/15/20/25/30 coexpressed genes with the highest correlation to each “seed” gene. In addition, the networks were further expanded using HPRD (vers. Release9_041310) PPI information. We investigated genes with PPIs with each “seed” gene and included them in the network. In total, we generated 6 × 5 = 30 expression-based networks (Fig. 1 **[bottom]**).

### Identification of subnetworks that were positively or negatively enriched in height-related genes

**Fig. 5:**
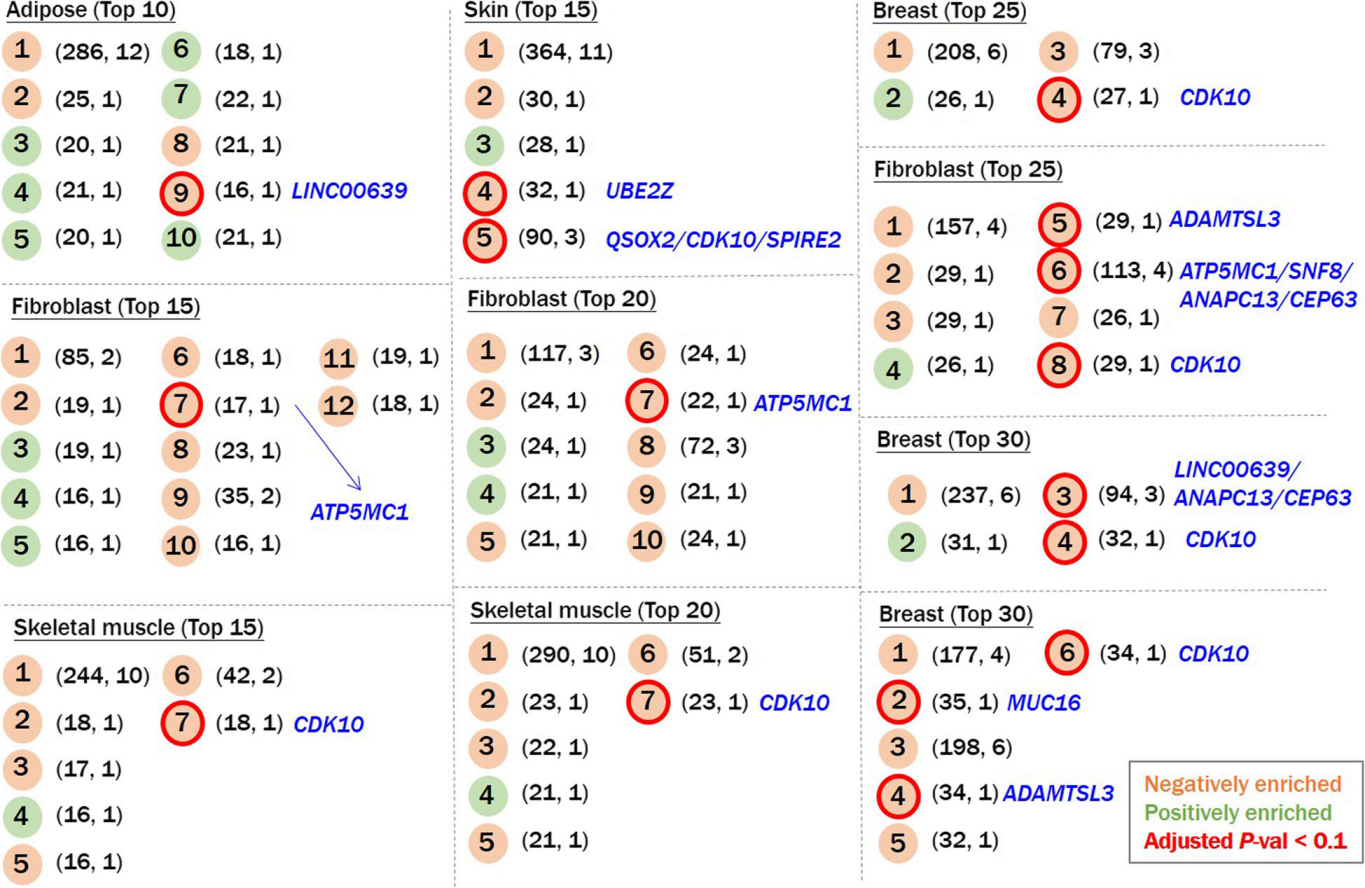
Brief view of networks that contained at least one significant subnetwork. The left number in the parentheses indicates the gene size of the subnetwork; and the right number in the parentheses indicates the number of “seed” genes within the subnetwork

To clarify the gene (integrated as a network)-phenotype relationship, we leveraged differentially expressed data related to the height from Taylor et al. [28] and performed a subnetwork-based GSEA. In the tissue-specific networks, each “seed” gene was linked with coexpression genes and/or PPI genes to form a subnetwork, which was possibly merged into a larger subnetwork if it contained at least one identical gene member with another subnetwork. For each amalgamated subnetwork, we conducted the GSEA (permutation no. = 99,999) by incorporating differential expression information, i.e., genes’ beta-coefficient statistics to the height. Significantly enriched (adjusted *p* < 0.1) subnetworks were defined as “height-related subnetworks”. 16 height-related subnetworks across 10 (33.3%) of 30 networks were identified, with network sizes ranging 16~113, and the number of “seed” genes ranging one to four. Notably, all identified height-related subnetworks were inversely correlated (negatively enriched) with expressions of genes that were positively associated with height (Fig. 5).

### Drug repositioning to FSS by targeting height-related subnetworks

**Fig. 6:**
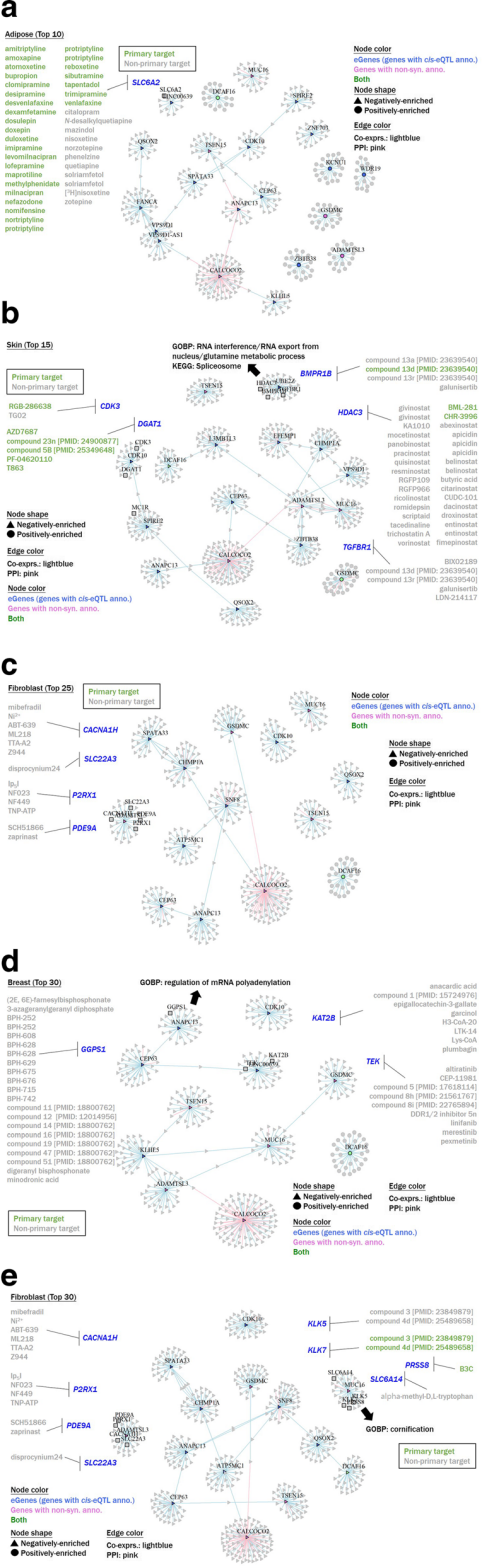
Network visualization of (**a**) adipose (top 10); (**b**) skin (top 15); (**c**) fibroblast (top 25); (**d**) breast (top 30); (**e**) fibroblast (top 30)

To integrate the direction of a drug’s effect on FSS into our pipeline, in other words, to elucidate drug-phenotype relationships, we incorporated (1) interaction data for ligands and targets (drug-gene relationship) from the Guide to PHARMACOLOGY database (vers. 2019.3) and (2) predefined gene-phenotype relationships (Fig.1 **[bottom]**). Given the Shefun pipeline, we determined that five of 30 networks (with seven different subnetworks spanning four tissue types) possessed repurposing potential, including (1) adipose (top 10) containing 39 ligand-gene pairs (Fig. 6a). In this network, *SLC6A2*, a norepinephrine transporter (NET) gene was identified as a potential drug target for SS repositioning. (2) Skin (top 15) containing 58 ligand-gene pairs (Fig. 6b). Two drug-targeted subnetworks were identified: one containing the drug-targeted genes *CDK3* and *DGAT1* and the other containing *BMPR1B*, *HDAC3,* and *TGFBR1*. (3) Fibroblast (top 25) containing 13 ligand-gene pairs (Fig. 6**c**). *CACNA1H*, *SLC22A3*, *P2RX1*, and *PDE9A* were identified as drug-targeted genes in this network. (4) Breast (top 30) containing 40 ligand-gene pairs (Fig. 6d) and drug-targeted genes such as *GGPS1*, *KAT2B* and *TEK*. (5) And, fibroblast (top 30) containing 19 ligand-gene pairs (Fig. 6e). In this network, two subnetworks were found to be potential candidates for drug repurposing, with one subnetwork containing the drug-targeted genes *KLK5*, *KLK7*, *PRSS8*, and *SLC6A14* and the other subnetwork containing *CACNA1H*, *P2RX1*, *PDE9A*, and *SLC22A3*. Therefore, these drugs/ligands could be candidates for further investigation. Given that some of the genes from the ligand-gene pairs that we identified might not be the primary target of the specific ligands, and might thus indicate possible safety issues, we therefore annotated information of “primary target” or “non-primary target” for each ligand-gene pair. This information may assist in the future prioritization of drugs/ligands for FSS repositioning.

### Pathways and biological processes over-representing drug-targeted subnetworks

For height-related subnetworks that contained the drug-targeted gene(s), we conducted GO BP terms and KEGG pathway ORA (Additional file 11**: Table S6**). The significant (with an FDR of < 0.1) BP terms and pathways are illustrated in Fig. 6a-e. For the skin (top 15), a subnetwork centered on *UBE2Z* (a “seed” gene) showed significant enrichment in RNA interference, RNA export from nuclei, glutamine metabolic process terms, and the spliceosome pathway (Fig. 6b). Another subnetwork (centered on *ANAPC13*) of the breast (top 30) also showed significant enrichment in the regulation of mRNA polyadenylation (Fig. 6d). In addition, a *MUC16*-centered subnetwork in the fibroblast (top 30) network showed significant enrichment in the cornification term (Fig. 6e).

## Discussion

In this work, we integrated several biological resources to prioritize FSS-related genetic variants and identified candidate druggable genes for FSS. Using a bioinformatics pipeline, we first annotated FSS-related variants and mapped those variants to genes (in the SNP-heuristic part). Next, we conducted gene-based annotations and prioritized genes in a network-based manner (in the expression-based functional unifying network part). As a result of this study, we reported five candidate networks for drug repositioning comprised of seven unique FSS-related genes (“seed” genes) including *LINC00639*, *CDK10*, *SPIRE2*, *QSOX2*, *ADAMTSL3*, *ANAPC13,* and *CEP63*. Overall, we identified 17 unique druggable genes.

Some of the determined druggable genes were reported to be directly associated with SS according to the Human Phenotype Ontology (HPO; the identity of SS: HP:0004322) and Gene-Disease Associations (GAD) databases, as exemplified by *SLC6A2* [30], a member of the Na^+^:neurotransmitter symporter family, which is targeted by some antipsychotic agents. Likewise, *BMPR1B*, a member of the bone morphogenetic protein (BMP) receptor family of transmembrane serine/threonine kinases, which belongs to the transforming growth factor (TGF)-β superfamily, was reported to be associated with acromesomelic dysplasia [31]. It is noteworthy that the BMP and TGF-β signaling pathways were suggested to play central roles in human growth, and hence are linked to the mechanism of the development of SS [32, 33]. *TGFBR1*, a gene that forms a heteromeric complex with the TGFBR2 protein, was also identified as a drug target of several TGF-β inhibitors for FSS repositioning in this study.

Additionally, we identified a number of druggable genes that may interact with known SS-related genes, despite they themselves are lacking of known associations with FSS, including *CDK3* (which interacts with *CABLES1*), *TGFBR1* (which interacts with *TGFB3*), *PDE9A* (which interacts with *HPRT1*), *TEK* (which interacts with *PIK3R1*), and *KLK7* (which interacts with *CDSN*). These genes were considered to be “indirectly” linked to FSS and might have potential to serve as targets for repurposing.

Furthermore, our results demonstrated several biologically meaningful gene clusters in drug repositioning for FSS: two groups of genes were related to the development biology pathway: one is a subnetwork in the network of “breast” (top 30), which contains *GGPS1*, *KAT2B,* and *TEK*. Specifically, *TEK* may interact with the SS-related gene, *PIK3R1*, which codes an enzyme that phosphorylates the 3′ position of the inositol ring of phosphatidylinositol [34]. *KAT2B*, a gene that associated with p300/CBP, mediates *PLK4* acetylation and thus acts as a negative regulator of centrosome amplification [35]. Notably, *PLK4* is also an SS-related gene. Impotyantly, we identified several acetyltransferase inhibitors that may target *KAT2B,* including anacardic acid, garcinol, plumbagin, and so on. The other gene cluster was located in the network of “fibroblast” (top 30), which contains *KLK5*, *KLK7*, *PRSS8*, and *SLC6A14*. In addition, *GGPS1*, a member of the prenyltransferase family, which encodes an enzyme that catalyzes the synthesis of geranylgeranyl diphosphate from farnesyl diphosphate and isopentenyl diphosphate, was associated with osteogenesis imperfecta. In addition, *GGPS1* was also reported to be correlated with the bone mineral density [36] and atypical femoral fractures [37]. In this study, we identified bisphosphonates that may target *KAT2B*. In addition, B3C, an activator of the epithelial sodium channel ENa, may target *PRSS8*. In short, we revealed several promising drugs, providing reasonable druggable gene clusters for FSS based on this genomic interrogation platform.

Nevertheless, we discovered two similar subnetworks in the “fibroblast” (top 25) and “fibroblast” (top 30), which contained druggable genes (*CACN1H*, *SLC22A3*, and *P2RX1*) that implicated in cation (calcium) homeostasis regulation, however, these genes have no clear connection to SS or FSS. Interestingly, a gene belonging to the above-mentioned subnetworks, *PDE9A*, is able to interact with *HPRT1*, which encodes an enzyme that is crucial for the generation of purine nucleotides through the purine salvage pathway, and is thus associated with SS. Therefore, our analysis may unearth previously unknown mechanisms/pathways of FSS which in turn, provides new insights for drug repositioning. Obviously, the findings need further rigorous experiments for validation.

The genome-wide scale association analysis that scanned the entire genome without bias provided an unprecedented opportunity for drug repurposing by linking disease indications with druggable genes, i.e.*,* “genetics-driven genomic drug discovery” [22, 38, 39], which is exemplified by the identification of *PCSK9* for the treatment of hypercholesterolemia [40]. We thus postulated that our “FSS-associated variants” should be subjected to a drug-repositioning analysis. We, therefore, leveraged the Guide to PHARMACOLOGY database to identify potential therapeutic agents that were initially developed for other diseases that may be repurposed to alleviate FSS. In addition, we showed the plausibility of drug target identification by using genomic approaches.

However, we noted several limitations. First, in GWAS part, false positives associations may not be excluded due to small power of current study. Second, further functional investigations are needed to validate the candidate drug targets identified by our annotation-based analytical pupeline. Third, the affinity and specificity of drugs that target SS-related genes may differ. Further experiments are required to select suitable drugs. Fourth, some druggable genes (e.g.*, SLC6A2*, *CDK3*, and *TEK*) were the targets of antipsychotic/anticancer agents, which may generally lead to more-severe adverse events. Therefore, in order to balance the risk and benefits, we emphasize that the genes targeted by safer agents should initially be prioritized to assess their clinical potential for repositioning to FSS.

## Conclusions

In summary, we prioritized seven candidate FSS-related genes (*LINC00639*, *CDK10*, *SPIRE2*, *QSOX2*, *ADAMTSL3*, *ANAPC13*, and *CEP63*) and 17 genes (*SLC6A2*, *CDK3*, *DGAT1*, *BMPR1B*, *HDAC3*, *TGFBR1*, *CACNA1H*, *SLC22A3*, *P2RX1*, *PDE9A*, *GGPS1*, *KAT2B*, *TEK*, *KLK5*, *KLK7*, *PRSS8*, and *SLC6A14*) for drug repurposing. Among them, drugs targeting *DGAT1*, *HDAC3*, *PDE9A*, *GGSP1*, *KAT2B*, *KLK5*, *KLK7*, *PRSS8*, and *SLC6A14* were recommended for repurposing not only due to the consideration of plausible mechanistic explanations but also after taking safety issues into evaluation. This study provides insights for understanding the pathophysiology of FSS and thereby conferring new approaches for drug discovery. Finally, our study demonstrated the power of comprehensive genomic interrogation in drug discovery for human diseases.

## Supplementary information

**Additional file 1: Fig. S1.** Gender-specific (male [top] and female [bottom]) age distribution curves of FSS patients (in this study; red color with solid line) and normal Taiwanese population (grey and black colors with solid and dashed lines). (DOCX 165 kb)

**Additional file 2: Fig. S2.** Multidimensional scaling (MDS) results. MDS plots of cases (CA) and controls (CN) in this study with (top) or without (bottom) other population from the 1000 Genome Database. (DOCX 77 kb)

**Additional file 3: Fig. S3.** Genome-wide association screening results. Manhattan plot of single-nucleotide polymorphisms (SNPs) on autosomal chromosomes under the additive inheritance model. The red line shows the threshold of the genome-wide association screening (*p* < 10^− 4^). (DOCX 119 kb)

**Additional file 4: Fig. S4.** 309 of 1751 (17.65%) unique single-nucleotide polymorphisms (SNPs) with at least one active chromatin state segmentation (states 1~19) in the following 12 cells (with brain-related cells excluded): mesenchymal stem cell-derived adipocyte cultured cells, adipose-derived mesenchymal stem cell cultured cells, HSMM cell-derived skeletal muscle myotubes cell line, muscle satellite cultured cells, bone marrow-derived cultured mesenchymal stem cells, foreskin melanocyte primary cells skin 01, foreskin melanocyte primary cells skin 03, NHDF-Ad adult dermal fibroblast primary cells, breast variant human mammary epithelial cells (vHMEC), HMEC mammary epithelial primary cells, osteoblast primary cells, mesenchymal stem cell-derived chondrocyte cultured cells. (DOCX 554 kb)

**Additional file 5: Fig. S5.** *Cis*-expression qualitative trait loci (eQTLs) associated with the expression of 70 unique genes (*a.k.a.* eGenes). (DOCX 91 kb)

**Additional file 6: Table S1.** Top 88 genetic loci identified from the initial genome-wide association study (GWAS) screening of Taiwanese familial short stature (FSS). (DOCX 19 kb)

**Additional file 7: Table S2.** Single-nucleotide polymorphism (SNP)-based regional annotation. (DOCX 12 kb)

**Additional file 8: Table S3.** Summary of four single-nucleotide polymorphisms (SNPs) located in exonic regions. (DOCX 12 kb)

**Additional file 9: Table S4.** Non-synonymous single-nucleotide polymorphisms (SNPs). (DOCX 14 kb)

**Additional file 10: Table S5.** Tissues with enrichment (*p* < 0.1) of chromHMM-annotated single-nucleotide polymorphisms (SNPs) are shown (Note: brain-related tissues were excluded from the downstream analysis). (DOCX 14 kb)

**Additional file 11: Table S6.** Subnetwork statistics. (DOCX 13 kb)

## Data Availability

None.

## Abbreviations

ASN: Asian
BH: Benjamini-Hochberg
BMP: Bone morphogenetic protein
BP: Biological process
EAS: East Asian
eQTL: Expression quantitative trait locus
FDR: False discovery rate
FR: Fruchterman-Reingold
FSS: Familial short stature
GAD: Gene-Disease Associations
GO: Gene ontology
GSEA: Gene set enrichment analysis
GTEx: Genotype-Tissue Expression
GWAS: Genome-wide association study
HPO: Human Phenotype Ontology
HPRD: Human Protein Reference Database
KEGG: Kyoto Encyclopedia of Genes and Genomes
LD: Linkage disequilibrium
MAF: Minor allele frequency
NET: Norepinephrine transporter
ORA: Over-representation analysis
PCA: Principal component analysis
PPI: Protein-protein interaction
QC: Quality control
rhGH: Recombinant human growth hormone
RIN: RNA integrity number
SD: Standard deviation
SS: Short stature
TGF: Transforming growth factor
UTR: Untranslated region
