# Supplementary figures and images for: Genomic interrogation of familial short stature contributes to the discovery of the pathophysiological mechanisms and pharmaceutical drug repositioning
Source: J Biomed Sci. 2019 Nov 7;26:91. doi: 10.1186/s12929-019-0581-2 (PMC6836357; doi:10.1186/s12929-019-0581-2)

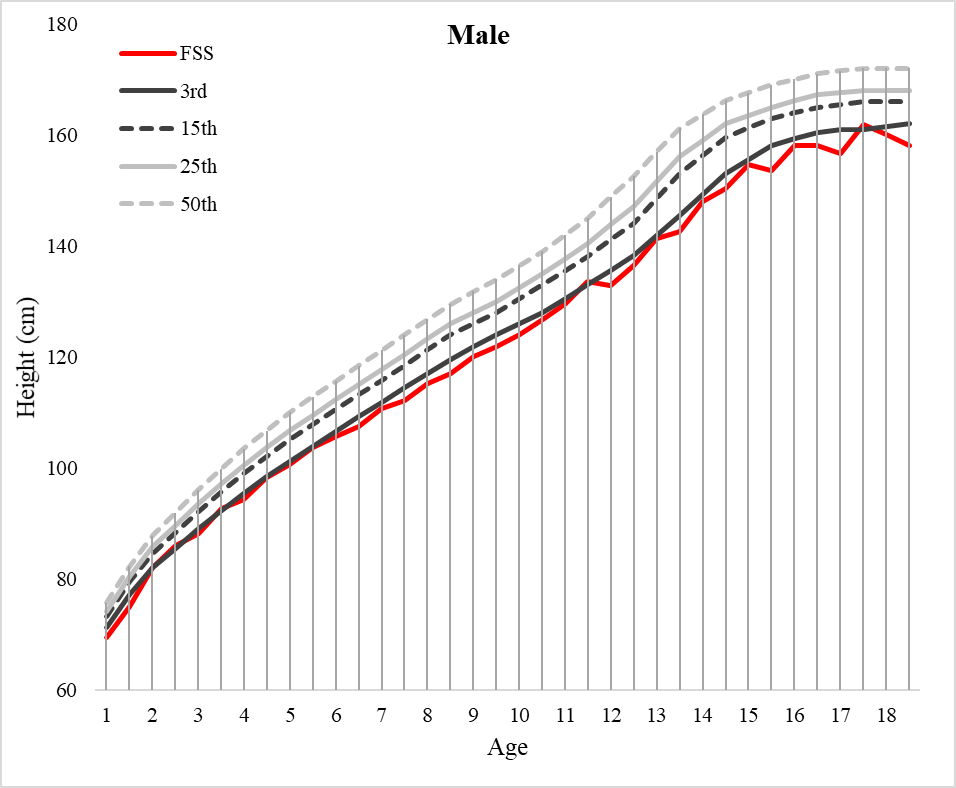


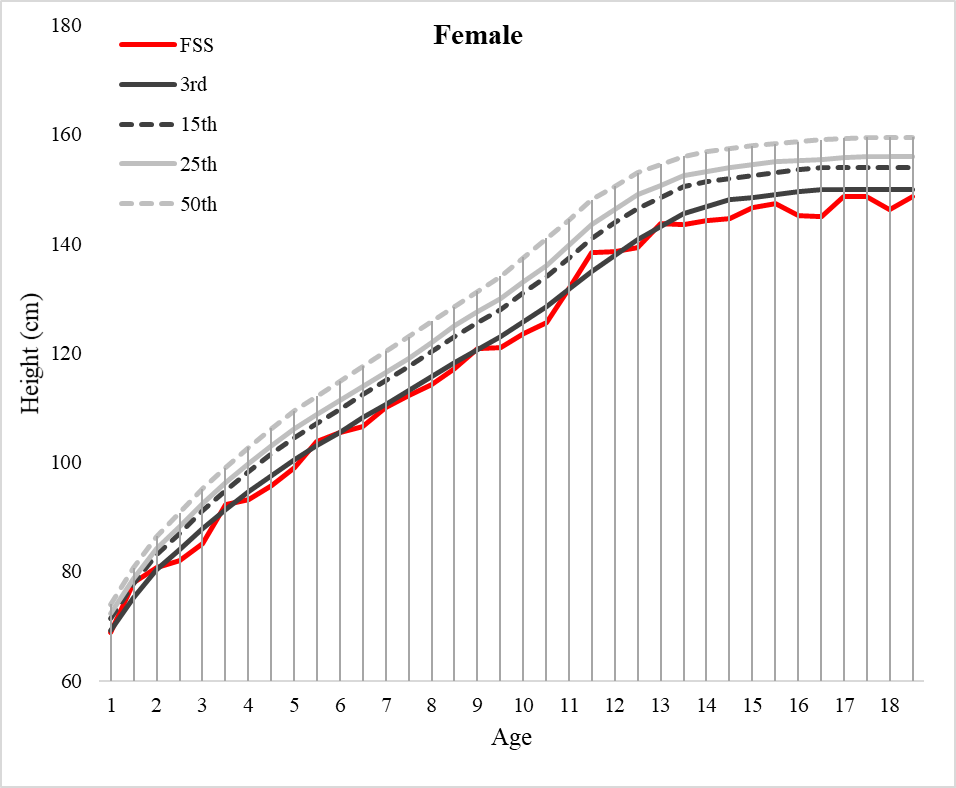

Supplement: Supplementary file 1 — Additional file 1: Fig. S1. Gender-specific (male [top] and female [bottom]) age distribution curves of FSS patients (in this study; red color with solid line) and normal Taiwanese population (grey and black colors with solid and dashed lines). (DOCX 165 kb) [file 12929_2019_581_MOESM1_ESM.docx]

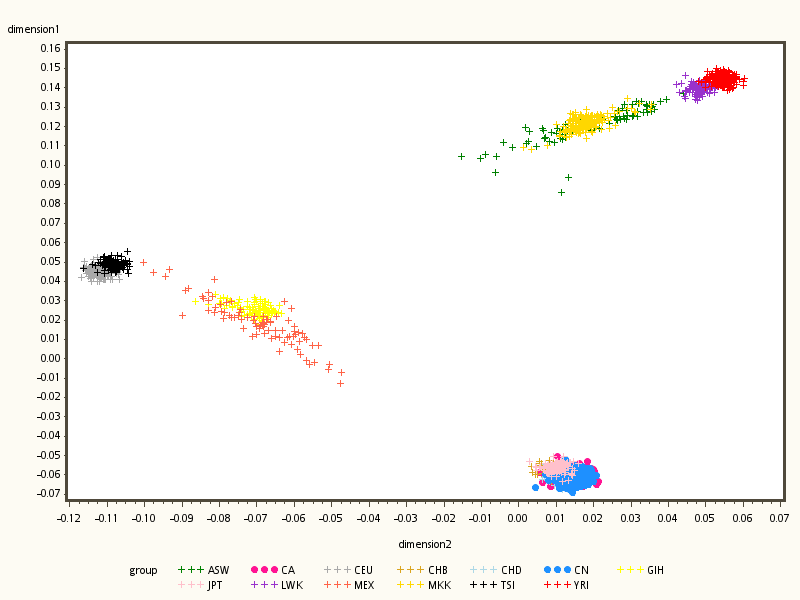


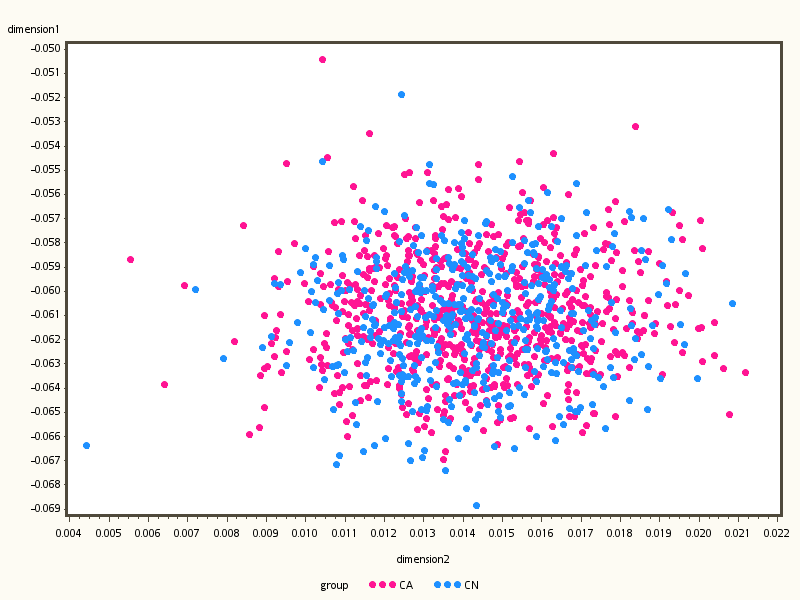

Supplement: Supplementary file 2 — Additional file 2: Fig. S2. Multidimensional scaling (MDS) results. MDS plots of cases (CA) and controls (CN) in this study with (top) or without (bottom) other population from the 1000 Genome Database. (DOCX 77 kb) [file 12929_2019_581_MOESM2_ESM.docx]

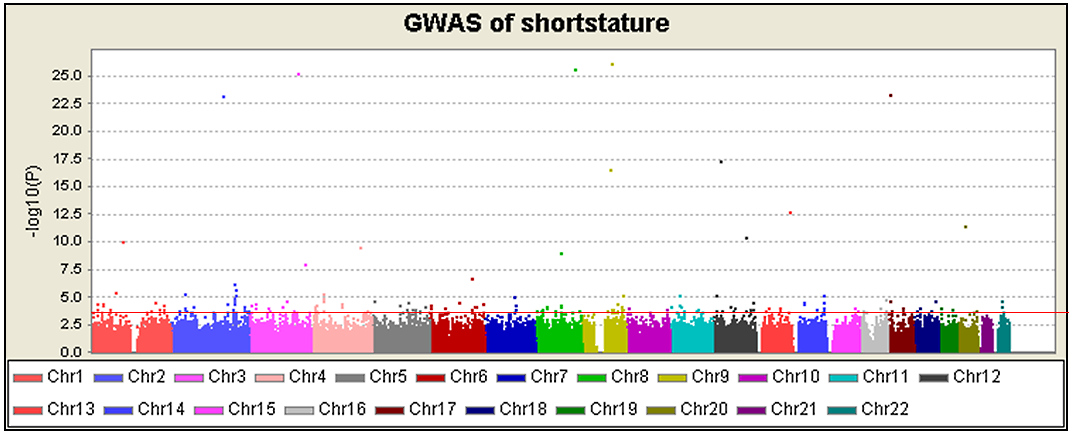

Supplement: Supplementary file 3 — Additional file 3: Fig. S3. Genome-wide association screening results. Manhattan plot of single-nucleotide polymorphisms (SNPs) on autosomal chromosomes under the additive inheritance model. The red line shows the threshold of the genome-wide association screening (p < 10− 4). (DOCX 119 kb) [file 12929_2019_581_MOESM3_ESM.docx]

**
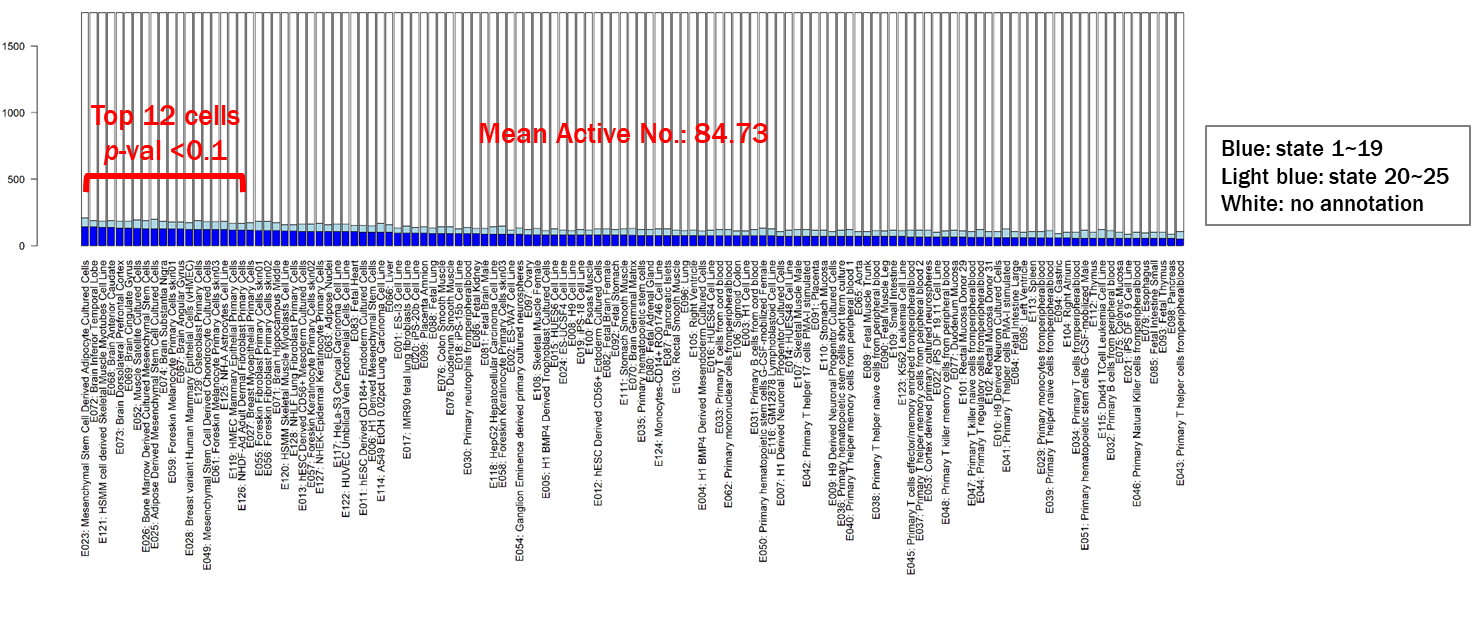
**

Supplement: Supplementary file 4 — Additional file 4: Fig. S4. 309 of 1751 (17.65%) unique single-nucleotide polymorphisms (SNPs) with at least one active chromatin state segmentation (states 1~19) in the following 12 cells (with brain-related cells excluded): mesenchymal stem cell-derived adipocyte cultured cells, adipose-derived mesenchymal stem cell cultured cells, HSMM cell-derived skeletal muscle myotubes cell line, muscle satellite cultured cells, bone marrow-derived cultured mesenchymal stem cells, foreskin melanocyte primary cells skin 01, foreskin melanocyte primary cells skin 03, NHDF-Ad adult dermal fibroblast primary cells, breast variant human mammary epithelial cells (vHMEC), HMEC mammary epithelial primary cells, osteoblast primary cells, mesenchymal stem cell-derived chondrocyte cultured cells. (DOCX 554 kb) [file 12929_2019_581_MOESM4_ESM.docx]

**
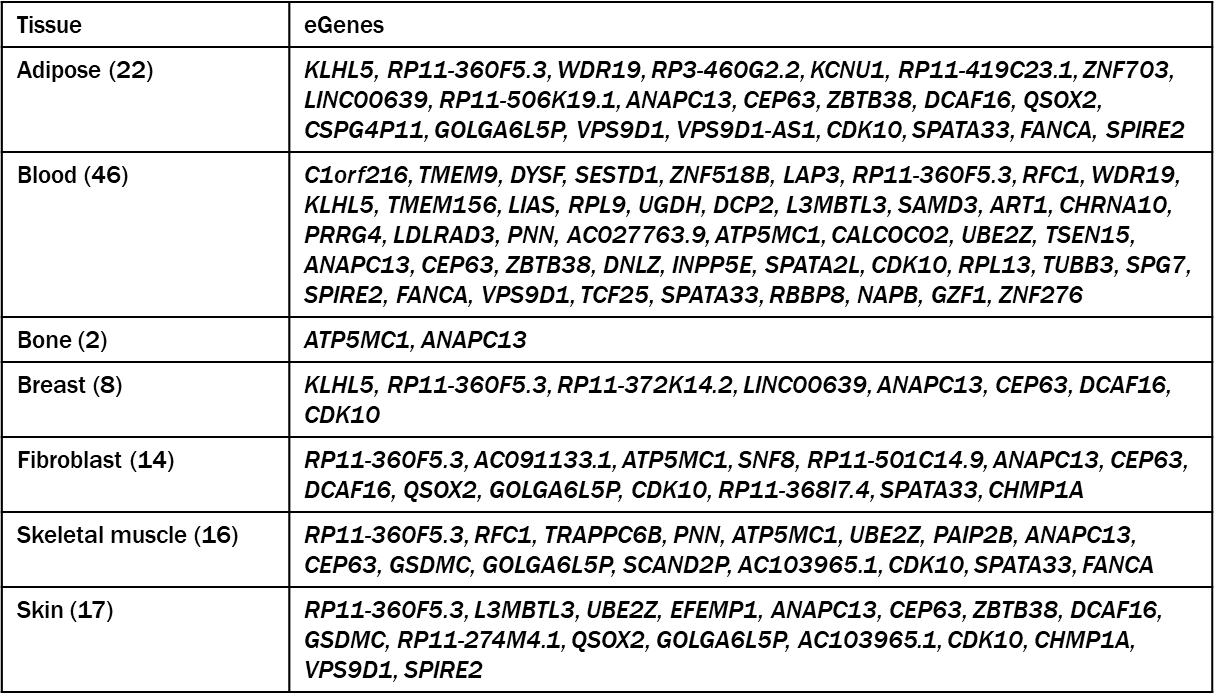
**

Supplement: Supplementary file 5 — Additional file 5: Fig. S5. Cis-expression qualitative trait loci (eQTLs) associated with the expression of 70 unique genes (a.k.a. eGenes). (DOCX 91 kb) [file 12929_2019_581_MOESM5_ESM.docx]
