# Supplementary material for: Genomic interrogation of familial short stature contributes to the discovery of the pathophysiological mechanisms and pharmaceutical drug repositioning
Source: J Biomed Sci. 2019 Nov 7;26:91. doi: 10.1186/s12929-019-0581-2 (PMC6836357; doi:10.1186/s12929-019-0581-2)
Supplement: Supplementary file 6 — Additional file 6: Table S1. Top 88 genetic loci identified from the initial genome-wide association study (GWAS) screening of Taiwanese familial short stature (FSS). (DOCX 19 kb) [file 12929_2019_581_MOESM6_ESM.docx]

| **Table S1.** The top 88 genetic loci identified from the initial GWAS screen of Taiwanese FSS. | | | | | |
| --- | --- | --- | --- | --- | --- |
| **dbSNP ID** | **Chromosome** | **Position** | **A1/A2** | **Gene Symbol** | ***p*-value** |
| rs75002618 | 1 | 18,146,409 | AG | *IGSF21* | 4.59E-05 |
| rs75856337 | 1 | 35,787,786 | AG | *AGO4* | 4.49E-05 |
| rs12059301 | 1 | 35,855,300 | TG | *AGO4* | 5.54E-05 |
| rs2152388 | 1 | 76,142,666 | TA | *ST6GALNAC3* | 4.44E-06 |
| rs822611 | 1 | 98,244,390 | AG | *LINC01776* | 1.12E-10 |
| rs10158900 | 1 | 201,125,443 | AG | - | 2.88E-05 |
| rs4653894 | 1 | 228,000,403 | TC | *WNT9A / WNT3A* | 5.63E-05 |
| rs6544781 | 2 | 45,051,655 | CG | - | 5.34E-06 |
| rs938659 | 2 | 45,736,761 | GA | *PRKCE* | 9.93E-05 |
| rs4428032 | 2 | 71,474,222 | AG | *DYSF* | 7.59E-05 |
| rs6731651 | 2 | 164,375,125 | AG | - | 6.05E-24 |
| rs10209887 | 2 | 179,240,039 | TC | *SESTD1* | 4.47E-05 |
| rs13417919 | 2 | 179,252,034 | CT | *SESTD1* | 4.31E-05 |
| rs17446700 | 2 | 200,255,505 | CT | - | 6.57E-07 |
| rs10931894 | 2 | 200,260,598 | GT | - | 2.57E-05 |
| rs12621262 | 2 | 200,271,396 | GA | - | 1.43E-05 |
| rs17630248 | 2 | 200,273,059 | CT | - | 3.62E-05 |
| rs78804475 | 2 | 204,209,362 | GA | *PARD3B / ICOS* | 7.61E-06 |
| rs12619767 | 2 | 204,211,467 | AG | *PARD3B / ICOS* | 2.23E-06 |
| rs16840902 | 2 | 204,224,539 | GA | *PARD3B / ICOS* | 2.23E-06 |
| rs7571816 | 2 | 232,212,354 | AG | *DIS3L2* | 8.35E-05 |
| rs1369117 | 3 | 10,699,638 | CT | *ATP2B2* | 5.29E-05 |
| rs12054035 | 3 | 25,227,730 | TC | - | 4.62E-05 |
| rs34436682 | 3 | 107,406,092 | AG | - | 7.40E-05 |
| rs59778278 | 3 | 121,090,749 | AC | *STXBP5L* | 2.27E-05 |
| rs16828530 | 3 | 157,932,964 | TC | - | 6.22E-26 |
| rs9290657 | 3 | 178,599,686 | CT | - | 9.91E-09 |
| rs7683687 | 4 | 10,521,221 | GA | *-* | 6.47E-05 |
| rs2286465 | 4 | 10,531,010 | GA | - | 8.96E-05 |
| rs7672919 | 4 | 17,795,832 | TG | *LOC105374509* | 4.80E-05 |
| rs12500509 | 4 | 39,061,994 | TG | *KLHL5* | 6.05E-06 |
| rs7658917 | 4 | 39,332,767 | TC | *RFC1* | 2.04E-05 |
| rs17584703 | 4 | 39,363,236 | CT | *RFC1* | 1.43E-05 |
| rs6857589 | 4 | 95,292,732 | TC | *UNC5C* | 6.76E-05 |
| rs6831808 | 4 | 95,292,777 | AG | *UNC5C* | 3.58E-05 |
| rs10028040 | 4 | 150,743,640 | TG | *LRBA* | 3.35E-10 |
| rs11743270 | 5 | 5,190,934 | GT | *ADAMTS16* | 2.34E-05 |
| rs1366595 | 5 | 89,045,356 | GA | *MEF2C* | 5.75E-05 |
| rs6899208 | 5 | 113,198,027 | TC | *MCC* | 3.09E-05 |
| rs1432929 | 5 | 166,806,051 | CT | - | 8.59E-05 |
| rs9405156 | 6 | 1,973,345 | CT | *GMDS* | 5.58E-05 |
| rs11965495 | 6 | 89,952,574 | TC | *BACH2* | 2.86E-05 |
| rs1415701 | 6 | 130,024,690 | AG | *L3MBTL3* | 2.26E-07 |
| rs1336369 | 6 | 140,640,515 | GA | - | 6.96E-05 |
| rs4394230 | 6 | 140,667,204 | TC | *LOC105378027* | 7.35E-05 |
| rs3804532 | 6 | 143,121,662 | AT | - | 7.65E-05 |
| rs3127409 | 6 | 166,120,196 | TC | - | 4.75E-05 |
| rs11767364 | 7 | 96,403,573 | GA | *LOC105375410* | 8.78E-06 |
| rs1207716 | 7 | 97,038,835 | CT | - | 5.67E-05 |
| rs10086016 | 8 | 36,990,191 | CT | - | 9.37E-05 |
| rs10955009 | 8 | 36,994,383 | AC | - | 8.43E-05 |
| rs9886374 | 8 | 79,396,368 | AG | *IL7* | 5.82E-05 |
| rs7014528 | 8 | 79,398,623 | GA | *IL7* | 7.39E-05 |
| rs1863593 | 8 | 80,536,906 | CT | - | 9.90E-10 |
| rs16900402 | 8 | 125,160,485 | GA | *NSMCE2* | 2.76E-26 |
| rs28786672 | 9 | 88,760,260 | CA | - | 3.45E-17 |
| rs7852806 | 9 | 93,846,345 | GT | *LOC101928014* | 8.65E-27 |
| rs16916289 | 9 | 111,675,744 | CT | - | 4.42E-05 |
| rs2416835 | 9 | 121,916,935 | CT | *TTLL11* | 6.94E-05 |
| rs12349999 | 9 | 126,017,533 | CT | - | 7.19E-06 |
| rs11254854 | 10 | 6,888,693 | TC | *LOC105376387* | 9.97E-05 |
| rs11023999 | 11 | 2,756,853 | AG | *KCNQ1* | 6.84E-05 |
| rs7945156 | 11 | 3,678,234 | GA | *NUP98* | 7.62E-05 |
| rs11032025 | 11 | 32,853,380 | AG | *PRRG4* | 7.70E-06 |
| rs10767971 | 11 | 32,874,118 | CT | - | 6.02E-05 |
| rs72901219 | 11 | 32,876,297 | CG | - | 6.39E-05 |
| rs4547071 | 11 | 36,030,588 | TC | *LDLRAD3* | 8.20E-05 |
| rs78430417 | 12 | 11,286,180 | AG | *-* | 6.73E-06 |
| rs2172912 | 12 | 26,045,337 | AG | *RASSF8* | 5.42E-18 |
| rs3816804 | 12 | 56,286,961 | TC | *CS* | 6.65E-05 |
| rs12826453 | 12 | 102,567,076 | CT | *IGF1* | 4.16E-11 |
| rs10444453 | 12 | 126,621,615 | TC | - | 3.34E-05 |
| rs9520911 | 13 | 108,501,255 | GA | *MYO16* | 2.34E-13 |
| rs12879229 | 14 | 39,027,867 | AG | - | 3.40E-05 |
| rs4902308 | 14 | 39,029,954 | AC | *SEC23A* | 3.84E-05 |
| rs17097800 | 14 | 98,791,403 | TC | *BCL11B* | 9.57E-05 |
| rs1257636 | 14 | 99,011,850 | AG | *BCL11B* | 3.50E-05 |
| rs4905794 | 14 | 99,013,332 | GA | *BCL11B* | 6.91E-06 |
| rs8055182 | 16 | 86,793,922 | AG | - | 1.58E-05 |
| rs311753 | 17 | 6,940,165 | GA | - | 2.17E-05 |
| rs17732181 | 17 | 6,957,288 | GT | - | 4.90E-24 |
| rs550510 | 17 | 48,849,253 | GA | *CALCOCO2* | 8.95E-05 |
| rs36084614 | 18 | 22,377,013 | TG | - | 9.90E-05 |
| rs7505525 | 18 | 70,369,400 | TC | - | 2.52E-05 |
| rs60104364 | 19 | 8,932,507 | AC | *MUC16* | 8.73E-05 |
| rs4815179 | 20 | 23,162,329 | CT | *LOC100505664* | 4.01E-12 |
| rs6005363 | 22 | 27,293,529 | TC | *MIAT / MN1* | 7.12E-05 |
| rs13054961 | 22 | 27,296,093 | AG | *MIAT / MN1* | 2.26E-05 |
| The top 88 SNPs (*p*–value <1×10^-4^) are identified by the genome-wide association study (GWAS) between FSS cases and controls under the additive inheritance model. These SNPs are ordered by the chromosome and position.  SNP, single nucleotide polymorphism; A1, allele 1 (minor-allele based the defined control whole samples); A2, allele 2 (major-allele based the defined control whole samples).  The positions are based on the NCBI GRCh38 version. Gene is identified based on the gene containing the SNP or the closest gene (within 100 kb up- or downstream) to the SNP. | | | | | |
