# Supplementary material for: Genomic interrogation of familial short stature contributes to the discovery of the pathophysiological mechanisms and pharmaceutical drug repositioning
Source: J Biomed Sci. 2019 Nov 7;26:91. doi: 10.1186/s12929-019-0581-2 (PMC6836357; doi:10.1186/s12929-019-0581-2)
Supplement: Supplementary file 7 — Additional file 7: Table S2. Single-nucleotide polymorphism (SNP)-based regional annotation. (DOCX 12 kb) [file 12929_2019_581_MOESM7_ESM.docx]

| **Table S2.** Single nucleotide polymorphism (SNP)-based regional annotation. | |
| --- | --- |
| **SNP location** | **No.** |
| Exonic | 4 (3.28%) |
| Intronic | 53 (43.44%) |
| ncRNA intronic | 5 (4.10%) |
| 5’ UTR | 0 (0.00%) |
| 3’ UTR | 1 (0.82%) |
| Intergenic | 58 (47.54%) |
| Upstream | 0 (0.00%) |
| Downstream | 0 (0.00%) |
| w/o annotation | 1 (0.82%) |
| Total | 122 (100.00%) |
