# Supplementary material for: Genomic interrogation of familial short stature contributes to the discovery of the pathophysiological mechanisms and pharmaceutical drug repositioning
Source: J Biomed Sci. 2019 Nov 7;26:91. doi: 10.1186/s12929-019-0581-2 (PMC6836357; doi:10.1186/s12929-019-0581-2)
Supplement: Supplementary file 8 — Additional file 8: Table S3. Summary of four single-nucleotide polymorphisms (SNPs) located in exonic regions. (DOCX 12 kb) [file 12929_2019_581_MOESM8_ESM.docx]

| **Table S3.** Summary of four SNPs located in exonic regions. | | | |
| --- | --- | --- | --- |
| **dbSNP ID** | **Gene** | **Functional type** | **a.a. change^a^** |
| rs11032025 | PRRG4 | Synonymous | P178P |
| rs550510 | CALCOCO2 | Non-synonymous | G68E |
| rs1046934 | TSEN15 | Non-synonymous | Q59H |
| rs4842838 | ADAMTSL3 | Non-synonymous | V661L |
| ^a^Amino acid. | | | |
