# Supplementary material for: Genomic interrogation of familial short stature contributes to the discovery of the pathophysiological mechanisms and pharmaceutical drug repositioning
Source: J Biomed Sci. 2019 Nov 7;26:91. doi: 10.1186/s12929-019-0581-2 (PMC6836357; doi:10.1186/s12929-019-0581-2)
Supplement: Supplementary file 9 — Additional file 9: Table S4. Non-synonymous single-nucleotide polymorphisms (SNPs). (DOCX 14 kb) [file 12929_2019_581_MOESM9_ESM.docx]

| **Table S4.** Non-synonymous SNPs. | | | | | | | | |
| --- | --- | --- | --- | --- | --- | --- | --- | --- |
| **dbSNP ID** | **Chr** | **Pos** | **Ref** | **Alt** | **Exonic func.** | **a.a.^a^ change** | **Query SNP** | **R-squared** |
| rs550510 | 17 | 46926615 | G | A | nonsynonymous | *CALCOCO2*:NM_001261395:exon4:c.G203A:p.G68E | rs550510 | 1 |
| rs11670318 | 19 | 9056982 | G | A | nonsynonymous | *MUC16*:NM_024690:exon3:c.C30464T:p.T10155I | rs60104364 | 0.904543 |
| rs2274432 | 1 | 184020945 | G | A | nonsynonymous | *TSEN15*:NM_001127394:exon1:c.G56A:p.G19D | rs1926872 | 1 |
| rs1046934 | 1 | 184023529 | A | C | nonsynonymous | *TSEN15*:NM_001127394:exon2:c.A177C:p.Q59H | rs1926872 | 1 |
| rs2274432 | 1 | 184020945 | G | A | nonsynonymous | *TSEN15*:NM_001127394:exon1:c.G56A:p.G19D | rs1046934 | 1 |
| rs1046934 | 1 | 184023529 | A | C | nonsynonymous | *TSEN15*:NM_001127394:exon2:c.A177C:p.Q59H | rs1046934 | 1 |
| rs7690457 | 4 | 17805379 | G | A | nonsynonymous | *DCAF16*:NM_017741:exon3:c.C386T:p.T129I | rs16895802 | 0.902414 |
| rs4144738 | 8 | 130760850 | A | G | nonsynonymous | *GSDMC*:NM_031415:exon14:c.T1424C:p.M475T | rs4733724 | 0.853008 |
| rs4144738 | 8 | 130760850 | A | G | nonsynonymous | *GSDMC*:NM_031415:exon14:c.T1424C:p.M475T | rs6470764 | 0.853008 |
| rs4842838 | 15 | 84582124 | G | T | nonsynonymous | *ADAMTSL3*:NM_001301110:exon16:c.G1981T:p.V661L | rs2401171 | 0.995094 |
| rs4144691 | 15 | 84539619 | C | G | nonsynonymous | *ADAMTSL3*:NM_001301110:exon9:c.C868G:p.L290V | rs2401171 | 0.970709 |
| rs4842838 | 15 | 84582124 | G | T | nonsynonymous | *ADAMTSL3*:NM_001301110:exon16:c.G1981T:p.V661L | rs10906982 | 0.995094 |
| rs4144691 | 15 | 84539619 | C | G | nonsynonymous | *ADAMTSL3*:NM_001301110:exon9:c.C868G:p.L290V | rs10906982 | 0.970709 |
| rs4842838 | 15 | 84582124 | G | T | nonsynonymous | *ADAMTSL3*:NM_001301110:exon16:c.G1981T:p.V661L | rs7183263 | 1 |
| rs4144691 | 15 | 84539619 | C | G | nonsynonymous | *ADAMTSL3*:NM_001301110:exon9:c.C868G:p.L290V | rs7183263 | 0.965775 |
| rs4842838 | 15 | 84582124 | G | T | nonsynonymous | *ADAMTSL3*:NM_001301110:exon16:c.G1981T:p.V661L | rs11259936 | 1 |
| rs4144691 | 15 | 84539619 | C | G | nonsynonymous | *ADAMTSL3*:NM_001301110:exon9:c.C868G:p.L290V | rs11259936 | 0.965775 |
| rs4842838 | 15 | 84582124 | G | T | nonsynonymous | *ADAMTSL3*:NM_001301110:exon16:c.G1981T:p.V661L | rs4842838 | 1 |
| rs4144691 | 15 | 84539619 | C | G | nonsynonymous | *ADAMTSL3*:NM_001301110:exon9:c.C868G:p.L290V | rs4842838 | 0.965775 |
| ^a^Amino acid. | | | | | | | | |
