# Supplementary material for: Genomic interrogation of familial short stature contributes to the discovery of the pathophysiological mechanisms and pharmaceutical drug repositioning
Source: J Biomed Sci. 2019 Nov 7;26:91. doi: 10.1186/s12929-019-0581-2 (PMC6836357; doi:10.1186/s12929-019-0581-2)
Supplement: Supplementary file 10 — Additional file 10: Table S5. Tissues with enrichment (p < 0.1) of chromHMM-annotated single-nucleotide polymorphisms (SNPs) are shown (Note: brain-related tissues were excluded from the downstream analysis). (DOCX 14 kb) [file 12929_2019_581_MOESM10_ESM.docx]

| **Table S5.** Tissues with enrichment (*p* <0.1) of chromHMM-annotated SNPs were showed (note: brain-related tissues were excluded from downstream analysis). | | | | | |
| --- | --- | --- | --- | --- | --- |
| Cell types | Active no. | Non-active no. | No anno.^a^ no | *Z*-score | One-sided *p* |
| E023: Mesenchymal Stem Cell Derived Adipocyte Cultured Cells | 143 | 64 | 1544 | 2.425201 | 0.007649946 |
| E072: Brain Inferior Temporal Lobe | 140 | 46 | 1565 | 2.300336 | 0.010714585 |
| E121: HSMM cell derived Skeletal Muscle Myotubes Cell Line | 139 | 46 | 1566 | 2.258715 | 0.011950569 |
| E068: Brain Anterior Caudate | 136 | 52 | 1563 | 2.13385 | 0.016427548 |
| E073: Brain Dorsolateral Prefrontal Cortex | 133 | 52 | 1566 | 2.008984 | 0.02226939 |
| E069: Brain Cingulate Gyrus | 131 | 54 | 1566 | 1.925741 | 0.027068356 |
| E052: Muscle Satellite Cultured Cells | 130 | 65 | 1556 | 1.884119 | 0.029774415 |
| E026: Bone Marrow Derived Cultured Mesenchymal Stem Cells | 127 | 60 | 1564 | 1.759254 | 0.039267165 |
| E025: Adipose Derived Mesenchymal Stem Cell Cultured Cells | 126 | 73 | 1552 | 1.717633 | 0.042931828 |
| E074: Brain Substantia Nigra | 126 | 56 | 1569 | 1.717633 | 0.042931828 |
| E059: Foreskin Melanocyte Primary Cells skin01 | 125 | 53 | 1573 | 1.676011 | 0.046868032 |
| E067: Brain Angular Gyrus | 125 | 52 | 1574 | 1.676011 | 0.046868032 |
| E028: Breast variant Human Mammary Epithelial Cells (vHMEC) | 123 | 49 | 1579 | 1.592767 | 0.055606183 |
| E129: Osteoblast Primary Cells | 123 | 66 | 1562 | 1.592767 | 0.055606183 |
| E049: Mesenchymal Stem Cell-Derived Chondrocyte Cultured Cells | 121 | 59 | 1571 | 1.509524 | 0.065582455 |
| E061: Foreskin Melanocyte Primary Cells skin03 | 121 | 60 | 1570 | 1.509524 | 0.065582455 |
| E125: NH-A Astrocytes Cell Line | 120 | 62 | 1569 | 1.467902 | 0.071065373 |
| E119: HMEC Mammary Epithelial Primary Cells | 119 | 50 | 1582 | 1.426281 | 0.076893672 |
| E126: NHDF-Ad Adult Dermal Fibroblast Primary Cells | 119 | 48 | 1584 | 1.426281 | 0.076893672 |
| ^a^Annotation. | | | | | |
