# Supplementary material for: Genomic interrogation of familial short stature contributes to the discovery of the pathophysiological mechanisms and pharmaceutical drug repositioning
Source: J Biomed Sci. 2019 Nov 7;26:91. doi: 10.1186/s12929-019-0581-2 (PMC6836357; doi:10.1186/s12929-019-0581-2)
Supplement: Supplementary file 11 — Additional file 11: Table S6. Subnetwork statistics. (DOCX 13 kb) [file 12929_2019_581_MOESM11_ESM.docx]

| **Table S6.** Subnetworks statistics. | | | | | | | | |
| --- | --- | --- | --- | --- | --- | --- | --- | --- |
| **Network** | **Subnetwork** | **Seed genes** | ***P*-value^a^** | ***P*_adj_^a^** | **ES^a^** | **NES^a^** | **GO BP term^b^** | **KEGG Term^b^** |
| skin (top 15) | cluster_4_size_32 | *UBE2Z* | 0.032322 | 0.080806 | -0.4161 | -1.53117 | RNA interference  RNA export from nucleus  glutamine metabolic process | Spliceosome |
| breast (top 30) | cluster_3_size_94 | *LINC00639*  *ANAPC13*  *CEP63* | 0.018819 | 0.037638 | -0.3183 | -1.47748 | regulation of mRNA polyadenylation |  |
| fibroblast (top 30) | cluster_2_size_35 | *MUC16* | 0.013054 | 0.039163 | -0.58043 | -1.71602 | cornification |  |
| ^a^Gene-set enrichment analysis with using height information from DL Taylor *et al*. ^b^Over-representation analysis. | | | | | | | | |
